# Association of plasma glial fibrillary acidic protein (GFAP) and Mediterranean diet

**DOI:** 10.1002/alz.090245

**Published:** 2025-01-09

**Authors:** E.M.S. Bandara, Samantha L Gardener, Steve Pedrini, Eugene Hone, Stephanie Rainey Smith, Ralph N Martins, Warnakulasuriya Mary Ann Dipika Binosha Fernando

**Affiliations:** ^1^ Edith Cowan University, Perth, Western Australia Australia; ^2^ Murdoch University, Perth, Western Australia Australia; ^3^ Alzheimer’s Research Australia, Perth, Western Australia Australia; ^4^ Australian Alzheimer’s Research Foundation, Perth, Western Australia Australia; ^5^ Australian Alzheimer's Research Foundation, Perth, Western Australia Australia; ^6^ Centre for Healthy Ageing, Murdoch University, Murdoch, Western Australia Australia; ^7^ Murdoch University, Murdoch, Western Australia Australia; ^8^ School of Psychological Science, University of Western Australia, Crawley, Western Australia Australia; ^9^ Department of Biomedical Sciences, Macquarie University, Macquarie Park, NSW Australia

## Abstract

**Background:**

Evidence suggests that diet may play a modifiable role in reducing Alzheimer’s disease (AD) risk. Higher adherence to the Mediterranean diet (MeDi) is linked to a lower incidence of mild cognitive impairment (MCI) and AD. Increasing evidence indicates the potential utility of a cytoskeletal protein found in astrocytes, plasma Glial Fibrillary Acidic Protein (GFAP), as a marker specific for AD pathogenesis. However, the relationship between plasma GFAP level and the MeDi has not been fully determined. This cross‐sectional study examined whether MeDi diet as a whole or the individual components, were associated with plasma GFAP levels in an Australian older adult cohort drawn from the larger Australian Imaging, Biomarkers and Lifestyle (AIBL) study of ageing.

**Methods:**

Cognitively unimpaired participants (n = 116) and individuals with MCI (n = 47) (mean age 75.37 ± 6.31; 44% male) completed a self‐reported food frequency questionnaire and had plasma GFAP levels measured using SIMOA platform. A score for the MeDi was generated for each individual. Linear regression models evaluated the association between plasma GFAP and the MeDi score, as well as individual MeDi components, including sex and age as covariates.

**Results:**

MeDi score was not associated with plasma GFAP level. However, for individual MeDi components, dairy intake was positively associated with GFAP level (β = 0.107±0.039, *p* = 0.007).

**Conclusions:**

This study is one of the first to report an association of plasma GFAP level with dietary components in an older adult cohort. The results suggest that dairy intake influences astrocyte reactivity. Further studies will include evaluation of plasma GFAP levels with additional dietary patterns, and individual dietary components to fully investigate the modifiable effect of diet on AD process.

